# Effectiveness of a Cardiovascular Management System on Triple Risk Factor Control in High-Risk Older Adults: Protocol for a Randomized Controlled Trial

**DOI:** 10.2196/84428

**Published:** 2025-12-09

**Authors:** Yan Wang, Lei Su, Jingyi Xiao, Xiaoying He, Hua Hong, Lin Xu

**Affiliations:** 1 Centre of Health Management, The First Affiliated Hospital, Sun Yat-sen University, Guangzhou, Guangdong, China; 2 Department of Geriatrics, The First Affiliated Hospital, Sun Yat-sen University, Guangzhou, Guangdong, China; 3 Department of Applied Health Sciences, University of Birmingham, Edgbaston, Birmingham, England, B15 2TT, United Kingdom, 86 20-87335523; 4 School of Public Health, Sun Yat-sen University, Guangzhou, China; 5 School of Public Health, University of Hong Kong, Hong Kong

**Keywords:** cardiovascular diseases, digital health, randomized controlled trial, risk factor control, medication adherence

## Abstract

**Background:**

Cardiovascular disease (CVD) remains a leading cause of morbidity and mortality in China. Although the National Basic Public Health Services Program provides annual health checkups for older adults, postscreening management of CVD risk factors such as hypertension, dyslipidemia, and diabetes is often inadequate. The CardioCare system is a digital cardiovascular management platform that integrates risk prediction, personalized health interventions, and continuous engagement to address these gaps.

**Objective:**

This trial aims to evaluate the effectiveness of the CardioCare system in improving triple risk factor control (simultaneous control of blood pressure, lipids, and glycemia) at 12 months among high-risk adults. Secondary objectives include assessing changes in estimated 10-year CVD risk, individual clinical parameters, medication adherence, lifestyle modification, system engagement, usability, satisfaction, and cost-effectiveness.

**Methods:**

This is a single-center, parallel-group, superiority randomized controlled trial with a 1:1 allocation ratio. A total of 300 adults aged ≥50 years with an estimated 10-year CVD risk of >10% and no previous CVD diagnosis will be recruited from the Health Management Center of the First Affiliated Hospital of Sun Yat-sen University in Guangzhou, China. Participants will be randomized to receive either the CardioCare system intervention or minimal usual care for 12 months. The intervention includes risk stratification and communication, weekly personalized SMS text messages or WeChat messages, and cardiologist oversight. The primary outcome is the proportion of participants achieving triple risk factor control at 12 months. Secondary outcomes include clinical, behavioral, usability, and economic measures. Analyses will follow the intention-to-treat principle, with multiple imputation for missing data.

**Results:**

This trial was funded on October 28, 2024. Recruitment is scheduled to begin in January 2026 and conclude in April 2026, with follow-up completed by June 2027. Data analysis will commence in mid-2027, and the main findings are expected to be published by the end of 2027. As of manuscript submission, recruitment has not yet started.

**Conclusions:**

This trial will provide robust evidence on the clinical effectiveness and cost-effectiveness of the CardioCare system in managing multiple cardiovascular risk factors among high-risk adults. The findings will inform the potential for scaling this intervention within health checkup centers and integrating it into national chronic disease management strategies.

## Introduction

The National Basic Public Health Services Program (NBPHSP) in China was launched in 2009 and has greatly improved access to annual health checkups for residents aged ≥65 years. This program aims to improve early detection and management of health issues among older adults, reaching approximately 173 million individuals, which constitutes approximately 12% of the population [1]. These checkups are designed to facilitate the early detection and management of various health conditions, particularly those prevalent in older adults, such as cardiovascular disease (CVD). Despite the extensive reach and potential benefits of this program, postcheckup management of health issues remains suboptimal [2].

Multiple systemic challenges contribute to these deficiencies. The fragmented nature of the health care system often results in poor coordination between primary care providers and specialists, leading to disjointed follow-up and inconsistent treatment [3]. Resource constraints, including shortages of trained personnel and limited funding, further limit the capacity for adequate postcheckup monitoring and support. Low patient engagement is another barrier; many patients fail to adhere to recommended treatments or lifestyle changes, diminishing the effectiveness of follow-up care [4,5]. Moreover, many individuals lack the necessary understanding of their condition and treatment plans, preventing effective self-management. Geographic and economic constraints, long waiting times, and inadequate electronic health record integration contribute to ongoing care fragmentation.

Screening alone is insufficient to improve disease awareness or management [6,7]. Many older adults in China have limited ability to comprehend screening results or the actions required in response [8,9]. Psychological barriers such as fear, denial, or anxiety may further reduce willingness to engage in follow-up care [10]. Infrastructure limitations, high costs, and poor physician-patient communication compound the problem [11,12]. Collectively, these factors underscore the need for a more integrated, patient-centered approach to postscreening management of chronic diseases, particularly CVD.

In light of these persistent challenges (ie, fragmented care, low patient engagement, and insufficient follow-up mechanisms), there is a clear need for innovative, scalable solutions that can support long-term cardiovascular risk management within routine care pathways. The CardioCare system was developed in response to these needs. It integrates an established CVD risk prediction model for risk stratification with personalized digital health management strategies, including tailored reminders, targeted lifestyle and medication interventions, and continuous feedback loops. By delivering individualized guidance and monitoring through a digital platform, the CardioCare system aims to enhance adherence to treatment plans and promote sustained behavior change, thereby improving control of key CVD risk factors. However, whether the implementation of the CardioCare system in routine health checkup services improves CVD management has yet to be examined.

Therefore, we will conduct a randomized controlled trial (RCT) to evaluate the effectiveness of the CardioCare system in improving triple risk factor control, defined as the simultaneous control of blood pressure, lipids, and glycemia, at 12 months among high-risk older adults identified through routine health checkups. Secondary objectives are to (1) assess changes in estimated 10-year CVD risk and individual clinical parameters, including systolic blood pressure (SBP), low-density lipoprotein cholesterol (LDL-C), and glycated hemoglobin (HbA_1c_); (2) evaluate medication adherence, adoption of lifestyle modifications, and patient engagement with the system; (3) assess system usability and satisfaction among participants and health care providers; and (4) examine the potential cost-effectiveness of the intervention compared with minimal usual care.

## Methods

### Participants

**Figure 1.**
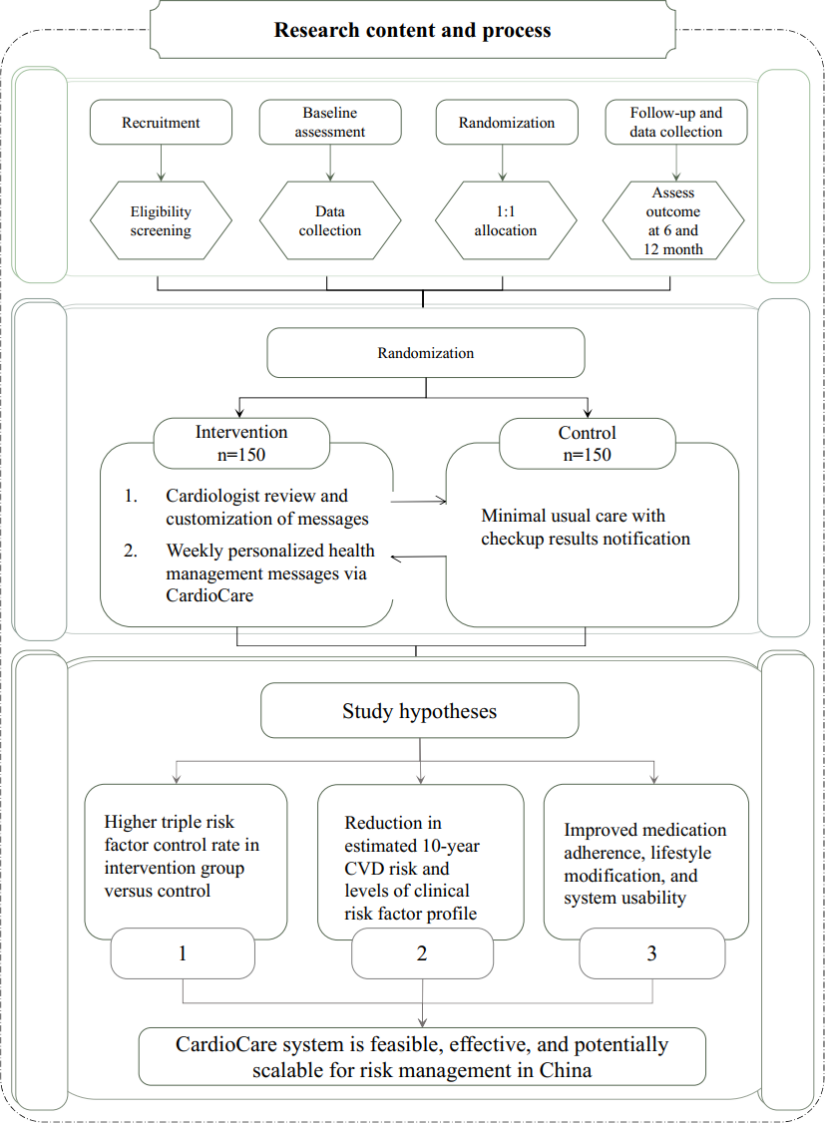
Study flowchart. CVD: cardiovascular disease.

This single-center, 2-arm, parallel-group RCT with a 1:1 allocation ratio will be conducted at the Health Management Center of the First Affiliated Hospital of Sun Yat-sen University in Guangzhou, China (Figure 1). The trial is designed within a superiority framework to determine whether the CardioCare system is more effective than minimal usual care in achieving triple risk factor control at 12 months among high-risk adults.

Eligible participants will be aged ≥50 years, have an estimated 10-year CVD risk greater than 10% calculated using the Guangzhou Biobank Cohort Study (GBCS) model [13], have no previous diagnosis of CVD, and own a mobile device capable of receiving messages from the CardioCare system. The GBCS model has been validated in a large urban Chinese cohort and is appropriate for identifying high-risk individuals in this population [13]. Participants must reside in Guangzhou for the duration of the study and provide written informed consent. Individuals will be excluded if they have severe comorbidities that preclude regular follow-up in the judgment of a study clinician based on medical record review, a life expectancy of less than 1 year, recent participation in similar CVD management programs, or any other condition judged by the investigators to interfere with safe participation.

### Random Allocation and Allocation Concealment

A statistician not involved in participant recruitment will generate the random allocation sequence using computer-generated random numbers. Randomization will be stratified by sex, with random block sizes ranging from 4 to 12 undisclosed to the recruitment team to ensure unpredictability. Allocation concealment will be maintained using sequentially numbered opaque, sealed envelopes prepared by staff independent of the enrollment process.

Research assistants will identify eligible participants, send invitations, and obtain informed consent, after which the next available envelope will be opened to reveal allocation. Personnel enrolling participants will not have access to the allocation sequence.

Participants and care providers will not be blinded due to the nature of the intervention. Outcome assessors and statisticians will remain blinded to allocation. Group codes will be masked during analysis, and the randomization key will only be revealed after the primary analysis is completed. No emergency unblinding procedures are required given the nonpharmacological nature of the intervention.

### Intervention and Control

The CardioCare system uses a structured message-tailoring algorithm that integrates clinical data, participant risk profiles, and previous engagement records to personalize message content and frequency. Cardiologists oversee message template development and conduct monthly reviews to ensure clinical relevance and appropriateness. Participant engagement is continuously monitored through automated system logs, which record message delivery and interaction. Predefined engagement thresholds trigger alerts to research staff for follow-up support. This operational framework follows the Template for Intervention Description and Replication guidance for intervention specification, ensuring reproducibility and transparency in intervention delivery.

The intervention group will receive a personalized intervention from the CardioCare system for 12 months. This system integrates CVD risk stratification using the GBCS model with tailored weekly SMS text messages or WeChat messages covering medication adherence, diet, exercise, and follow-up reminders. Messages will be reviewed by a cardiologist before dispatch and customized based on each participant’s clinical profile and previous engagement. Message types will vary in intensity and content (eg, motivational, educational, or reminder based) depending on previous adherence and risk level. All messages are logged automatically, and participant engagement (eg, message reads, replies, and link clicks) is continuously monitored. Participants with declining engagement will be flagged for follow-up by the research assistant team, ensuring responsiveness. Cardiologist review takes place monthly or upon significant clinical change using an internal dashboard linked to participant records.

The control group will receive minimal usual care, consisting of notification of health checkup results and general recommendations to seek outpatient follow-up if indicated. After completing the 12-month follow-up, control group participants will be offered access to the CardioCare system. Participants may discontinue the intervention at any time or if advised by the study physician. No restrictions will be placed on concomitant medical care.

### Outcomes

The primary outcome is the proportion of participants achieving simultaneous control of blood pressure, lipids, and glycemia at 12 months, defined as SBP of <130 mm Hg and diastolic blood pressure of <80 mm Hg, LDL-C of <2.6 mmol/L, fasting plasma glucose of <7.0 mmol/L, and HbA_1c_ of <7% (Table 1). Secondary outcomes include (1) clinical measures (changes in estimated 10-year CVD risk [GBCS model]; mean changes in SBP, LDL-C, triglycerides, fasting plasma glucose, and HbA_1c_; and the proportion achieving control of individual risk factors at 12 months), (2) behavioral measures (medication adherence and adoption of lifestyle modifications), (3) system-level outcomes (participant engagement with the CardioCare system and usability and satisfaction among participants and physicians), and (4) economic outcomes (incremental cost-effectiveness expressed as cost per participant achieving triple risk factor control and per quality-adjusted life year [QALY] gained; Table 1).

Medication adherence and lifestyle modifications (diet, physical activity, and smoking) will be assessed using structured questionnaires adapted from previously validated tools applied in chronic disease studies in Chinese populations. Measurements for all primary and secondary outcomes will be taken at baseline and 6 and 12 months after enrollment. To assess feasibility and acceptability, we will evaluate (1) recruitment rate (participants enrolled and approached); (2) retention rate at 12 months; (3) system engagement metrics, including number of messages opened and responded to; and (4) participant-reported usability and satisfaction at 12 months. Usability will be assessed using the System Usability Scale, and satisfaction will be measured using a 5-point Likert scale. These data will inform scalability potential and guide future implementation strategies. Quantitative feasibility metrics will be summarized descriptively, and comparisons between groups will be exploratory.

In addition to formal clinical and behavioral assessments at baseline and 6 and 12 months, the CardioCare system captures weekly engagement data, including message delivery, read status, interaction frequency, and responsiveness. These metrics will be used to construct engagement trajectories and assess temporal patterns of system use. The economic evaluation will be conducted from the health care system perspective. Direct medical costs will include personnel time, digital intervention costs (eg, system maintenance and messaging), outpatient visits, medications, and laboratory tests. Cost-effectiveness will be assessed through incremental cost-effectiveness ratios reported as cost per participant achieving triple risk factor control and per QALY gained.

**Table 1.** Outcome measure summary.

Outcome	Definition and measure	Time point	Assessment method
Primary outcome	Triple risk factor control (SBP^a^ of <130 mm Hg, DBP^b^ of <80 mm Hg, LDL-C^c^ of <2.6 mmol/L, and HbA_1c_^d^ of <7%)	12 months	Clinical measurements and laboratory assays
Secondary outcomes			
10-year CVD^e^ risk	Estimated via GBCS^f^ model	Baseline and 12 months	Model-based calculation
SBP, LDL-C, HbA_1c_, FPG^g^, and triglycerides	Individual clinical parameters	Baseline and 6 and 12 months	Hospital-certified laboratory and digital BP^h^ monitor
Medication adherence	Self-reported adherence (structured questionnaire)	6 and 12 months	Validated adherence scale
Lifestyle modification	Diet, physical activity, and smoking status	6 and 12 months	Structured behavioral questionnaire
System engagement	Frequency of interactions with the CardioCare system	Continuous	Digital system logs
Usability	Perceived ease of use	12 months	SUS^i^
Satisfaction	Overall satisfaction with the system	12 months	5-point Likert scale questionnaire
Cost-effectiveness	Cost per case of triple risk factor control and QALYs^j^ gained	Baseline and 12 months	Economic modeling using trial data

a SBP: systolic blood pressure.

b DBP: diastolic blood pressure.

c LDL-C: low-density lipoprotein cholesterol.

d HbA_1c_: glycated hemoglobin.

e CVD: cardiovascular disease.

f GBCS: Guangzhou Biobank Cohort Study.

g FPG: fasting plasma glucose.

h BP: blood pressure.

i SUS: System Usability Scale.

j QALY: quality-adjusted life year.

### Sample Size Estimation

The sample size was calculated to detect an absolute difference of 15 percentage points in the primary outcome (ie, 20% in the control group vs 35% in the intervention group), with 80% power and a 2-sided α of .05. This requires 126 participants per arm. Allowing for 15% attrition, the final target sample size is 300 participants (150 per group). This expected difference is based on previous studies in Chinese older adult populations showing triple risk factor control rates ranging from 15% to 25% under usual care and on effect sizes from similar multicomponent digital interventions, which have reported 10– to 20–percentage point improvements in risk factor control [14]. Recruitment will take place during routine health checkup visits, with eligible individuals identified from their health data and approached in person by a trained research assistant. Additional recruitment will be supported through study posters in the Health Management Center and announcements on the hospital’s WeChat platform.

### Data Collection and Management

Clinical and laboratory measurements will be collected at baseline and 6 and 12 months using standardized procedures. Blood pressure will be measured in duplicate using validated automated devices after 5 minutes of rest; the average reading will be recorded. Lipid profiles and glycemia will be measured in fasting venous samples analyzed at the hospital’s certified laboratory. Medication adherence will be evaluated using a self-administered online questionnaire. Lifestyle modification and system engagement data will be collected through structured questionnaires. All data will be entered into a secure, password-protected database, and automated range checks will be conducted. Regular audits will be conducted to ensure accuracy. Personal identifiers will be stored separately from trial data accessible only to authorized personnel.

### Statistical Analysis

All analyses will follow the intention-to-treat principle, with all randomized participants included in the group to which they were assigned. The primary outcome will be compared between groups using a chi-square test, with risk differences and 95% CIs calculated. Secondary continuous outcomes will be analyzed using mixed-effects models adjusting for baseline values. Missing data patterns will be assessed visually and statistically (eg, the Little missing completely at random test) to inform appropriate imputation models. Multiple imputation using chained equations will be used under the assumption of the data being missing at random, and sensitivity analyses will include complete-case and worst-case scenarios. Prespecified subgroup analyses will examine potential effect modification by sex, age group, and baseline CVD risk. These subgroup analyses are exploratory in nature and will not be powered to detect statistically significant differences. Findings will be interpreted descriptively and used to inform hypothesis generation for future trials.

QALYs will be estimated using utility weights derived from published data on health-related quality of life in Chinese adults. A within-trial cost-effectiveness analysis will be conducted over the 12-month intervention period. Sensitivity analyses will be conducted to examine the robustness of the incremental cost-effectiveness ratio to variations in key cost and outcome inputs. All analyses will be conducted using Stata (version 18.0; StataCorp). A 2-sided *P* value of <.05 will be considered statistically significant.

### Monitoring

A data monitoring committee comprising independent epidemiologists and cardiologists will oversee trial conduct, review adverse events, and provide recommendations regarding trial continuation. No interim analyses or formal stopping rules are planned. The trial will be monitored monthly by the study management team to ensure protocol adherence. The data monitoring committee will make recommendations regarding trial continuation or necessary modifications. Oversight procedures will follow International Council for Harmonisation of Technical Requirements for Registration of Pharmaceuticals for Human Use good clinical practice guidelines.

### Ethical Considerations

Ethics approval has been obtained from the institutional review board of the School of Public Health, Sun Yat-sen University (2024-110). Written informed consent will be obtained from all participants by trained research assistants. Protocol amendments will be submitted to the institutional review board and communicated to trial staff and, if necessary, participants. Confidentiality will be maintained by assigning unique study IDs, with personal information stored separately from trial data. After the trial, control participants will receive access to the CardioCare system for 12 months. Participants who experience harm related to trial participation will receive appropriate medical care and compensation according to institutional policy.

## Results

This trial was funded on October 28, 2024. Participant recruitment is scheduled to commence in October 2026 at the Health Management Center of the First Affiliated Hospital of Sun Yat-sen University and is expected to continue for approximately 9 months. Each participant will be followed up on for 12 months after randomization, with clinical assessments at baseline (0 months), 6 months, and 12 months. Data analysis is expected to begin shortly after the final 12-month assessments are completed. At the time of manuscript submission, recruitment has not yet commenced. Table 2 shows the trial schedule [15], with enrollment procedures at baseline; continuous delivery of the intervention up to 12 months; and timing of assessments at baseline and 6 and 12 months, with cost-effectiveness evaluated at baseline and 12 months.

**Table 2.** Participant timeline: schedule of enrollment, intervention, and assessments.

	Trial period			
	Enrollment		Postrandomization closeout	
	−3 to 0 months	Baseline	6 months	12 months
Enrollment				
Eligibility screen	✓			
Informed consent	✓			
Randomization		✓		
Intervention and comparator				
Intervention		✓	✓	
Comparator		✓	✓	
Risk communication by cardiologists		✓		
Demographics, medical history, anthropometrics, blood pressure, laboratory tests, 10-year CVD^a^ risk, and behavioral and psychosocial measures	✓	✓		
Primary outcome (triple risk factor control at 12 months [BP^b^, LDL-C^c^, FPG^d^, and HbA_1c_^e^]) and secondary outcomes (estimated 10-year CVD risk, individual clinical parameters, medication adherence, lifestyle modification, system engagement, usability, and satisfaction)			✓	✓
Cost-effectiveness (clinical assessments, laboratory tests, validated questionnaires, system logs, and economic analysis)				✓

a CVD: cardiovascular disease.

b BP: blood pressure.

c LDL-C: low-density lipoprotein cholesterol.

d FPG: fasting plasma glucose.

e HbA_1c_: glycated hemoglobin.

## Discussion

### Expected Findings

CVD remains the leading cause of morbidity and mortality in China, particularly among older adults. Although the NBPHSP has greatly expanded access to routine health checkups, postscreening management of chronic conditions such as hypertension, dyslipidemia, and diabetes is often suboptimal [16]. The CardioCare system was designed to address these gaps by integrating CVD risk prediction, personalized health management, and continuous digital engagement into existing health care workflows. This RCT will be the first to evaluate whether such a system can improve simultaneous control of blood pressure, lipids, and glycemia among high-risk older adults in China.

This RCT has several strengths. First, it addresses a critical gap in postcheckup care by focusing on triple risk factor control, a clinically meaningful composite end point closely linked to CVD outcomes. Second, the intervention is built on an established risk prediction model (the GBCS model) and combines risk communication and evidence-based lifestyle and medication adherence strategies with personalized digital communication. Third, integration into an existing health checkup center ensures feasibility in a real-world setting, and the trial includes cost-effectiveness analysis to inform potential scalability.

There are also potential limitations. This trial is conducted in a single urban health management center, which may limit generalizability to rural or resource-limited settings where infrastructure and cardiovascular risk management practices differ. However, as an initial trial of the CardioCare system, this setting enables evaluation under conditions of high intervention fidelity. Future studies will be needed to assess adaptations for broader populations, particularly in underresourced areas or settings with lower digital capacity. The reliance on self-reported measures for some behavioral outcomes, such as medication adherence and lifestyle modification, introduces the possibility of reporting bias. Engagement with the digital intervention may vary between participants, potentially influencing its effectiveness. In addition, while the triple risk factor control outcome is clinically relevant, it may be more challenging to achieve than improvements in individual risk factors, potentially underestimating the system’s benefit. If the trial demonstrates effectiveness, the CardioCare system could be scaled to other health checkup centers and integrated into national health programs, potentially improving chronic disease management for millions of older adults. Although mobile device access and digital communication literacy are eligibility requirements, these are unlikely to be significant sources of exclusion in the Chinese context, where smartphone ownership and WeChat use exceed 99% among Guangzhou residents, including those over the age of 50 years. Future research could explore adaptations for rural settings, automation of message delivery, integration with other chronic disease management modules, and long-term effects on CVD events and mortality. Finally, as the trial is conducted within a single health management center, there is a potential for contamination between intervention and control participants. Although the CardioCare intervention is delivered individually via private digital communication and participants do not engage in group sessions, the possibility of informal information sharing cannot be entirely excluded.

### Conclusions

This RCT will evaluate the effectiveness of the CardioCare system, a digital cardiovascular management platform, in achieving simultaneous control of blood pressure, lipids, and glycemia among high-risk adults identified through routine health checkups. By integrating established risk prediction with personalized health management and continuous digital engagement, the intervention aims to address critical gaps in postscreening care within the Chinese health care system. The findings will provide robust evidence on both the clinical impact and cost-effectiveness of the CardioCare system, informing its potential for large-scale implementation in health checkup centers and integration into national chronic disease prevention strategies. If effective, the CardioCare system could be scaled through existing national platforms such as the NBPHSP and align with priorities outlined in Healthy China 2030. Its digital format supports integration into routine primary care and health checkup services, potentially strengthening chronic disease management at a population level.

## Abbreviations

CVD: cardiovascular disease
GBCS: Guangzhou Biobank Cohort Study
HbA_1c_: glycated hemoglobin
LDL-C: low-density lipoprotein cholesterol
NBPHSP: National Basic Public Health Services Program
QALY: quality-adjusted life year
RCT: randomized controlled trial
SBP: systolic blood pressure
